# Dietary Factors, Time of the Week, Physical Fitness and Saliva Cortisol: Their Modulatory Effect on Mental Distress and Mood

**DOI:** 10.3390/ijerph19127001

**Published:** 2022-06-08

**Authors:** Lina Begdache, Saloumeh Sadeghzadeh, Paul Pearlmutter, Gia Derose, Pragna Krishnamurthy, Ahyeon Koh

**Affiliations:** 1 Health and Wellness Studies, Binghamton University, Binghamton, NY 13902, USA; 2 School of Management, Binghamton University, Binghamton, NY 13902, USA; ssadeghz@binghamton.edu; 3 Department of Biomedical Engineering, Binghamton University, Binghamton, NY 13902, USA; ppearlm1@binghamton.edu (P.P.); akoh@binghamton.edu (A.K.); 4 Department of Biological Sciences, Binghamton University, Binghamton, NY 13902, USA; gderose1@binghamton.edu; 5 Department of Integrative Neuroscience, Binghamton University, Binghamton, NY 13902, USA; pkrishn1@binghamton.edu

**Keywords:** time of the week, exercise, diet, saliva cortisol, mood, mental distress

## Abstract

Background: The purpose of the study was to assess the effect of diet quality and physical fitness on saliva cortisol, mood, and mental distress. These relationships were compared between a peak weekday (Wednesday) and a weekend day (Saturday) when mood may fluctuate. Methods: Forty-eight healthy college students participated in the study. Participants completed the Mood and Anxiety Symptom (MASQ) and Kessler Psychological Distress Scale 10 questionnaires on Wednesday and Saturday and recorded their diet for three days. Saliva was collected before and after a workout for cortisol extraction. Results: SA had significantly higher saliva cortisol levels post-workout but lower MASQ scores on Saturday (*p* < 0.05). There was a very significant association between MASQ scores on Wednesday (*p* = 0.005), which became less significant on Saturday. In addition, lower BMI values and high-fat consumption were associated with higher cortisol levels after exercise (*p* < 0.05). Conclusions: There is a strong link between dietary factors, cortisol levels, mood, and time of the week. In addition, our results suggest that saliva cortisol levels may not be directly linked to negative affect but are influenced by diet quality when mental distress exists. In addition, physical fitness may play a role in improving mood during weekends.

## 1. Introduction

Physical fitness is the product of regular exercise, which decreases the risk of several health conditions including mental health ailments. Regular exercise improves mental health through numerous advantageous biochemical and physiological adjustments [1]. In particular, cardiovascular exercise reduces symptoms of mental distress through several cellular adaptations [2,3], with some evidence suggesting that resistance exercise may bear the same effect as well [4]. In part, the beneficial effect of exercise is linked to physiological adaptations. Specifically, exercise stimulates the release of vascular endothelial growth factor (VEGF) and brain-derived neurotrophic factor (BDNF), which improve regional cerebral blood flow [5] that supports neurogenesis and neuroplasticity [5], respectively. Therefore, this increase in blood circulation coupled with enhancement in neurotransmission improves mental well-being. Exercise boosts brain serotonin and dopamine levels, which modulate positive mood and motivation, respectively [6,7]. An enhanced motivation has been linked to improvements in diet and lifestyle factors, which eventually promote mental well-being [8]. In addition, exercise moderates the hypothalamic–pituitary–adrenal (HPA) axis activity that synchronizes communication between key emotion-processing areas of the brain such as the limbic system, amygdala, and hippocampus. The HPA axis is vital in regulating homeostasis and the body’s natural response to stress. Because glucocorticoid receptors exist in nearly every tissue in the body, the stress response affects almost every organ system [9].

However, psychological stress impedes mental well-being, reduces the effort to exercise, and impairs muscle recovery [10]. These facts propose that exercising under stress may add to the mental distress etiology. Typically, low to moderate exercise lowers levels of the stress hormone, cortisol, which supports overall mental well-being. However, vigorous exercise has the opposite effect on cortisol levels and may exacerbate the stress response [11]. This fact suggests that athletes who are physically fit but practice daily for hours may experience heightened mental distress, which makes them an attractive population to study stress in relation to physical fitness. A systematic review of research addressing the mental health of elite athletes proposed that this population is susceptible to an assortment of mental health ailments due to their stressful careers [12]. Sports club and college athletes, competing at the national level, may have the extra burden of academic achievement, which may add pressure to their athletic experience. However, stress, mental health, and physical fitness may not possess a linear relationship. There are several dimensions to consider. A prominent one is the age group. Young adults are prone to stress due to the incomplete maturation of their prefrontal cortex. The latter controls emotions and impulses as well as several cognitive functions. In addition, there is an established gender difference in response to emotional stimuli [13]. Women are more likely to display psychological distress and eating disorders under stress when compared with men [14]. Recent evidence suggests that mental distress in women is associated with low-quality diet scores [15]. Correspondingly, the quality of the diet may influence levels of cortisol secretion [16] and may worsen mental toughness. An investigation into the diet quality of student-athletes reported high consumption of processed food with several micronutrient deficiencies [17]. These combined findings suggest that student-athletes who consume an imbalanced diet may experience higher levels of stress, thus exhibiting higher cortisol levels and mental distress. Therefore, general nutritional knowledge among athletes is crucial in improving diet quality and mental health. Consequently, the combination of physical fitness and a low-quality diet may not lead to an improvement in mental status. This is a topic in need of further research as most work ties physical fitness to mental well-being and a low-quality diet to poor mental health, but, to our knowledge, no reports investigated the mutual effects. The closest report by Strasser and Fuchs [11] eloquently outlines the role of exercise intensity on mental health and the effect of specific dietary factors on brain health. Additionally, it is crucial to account for the time of the week to complete the big picture, [18] as mood and dietary intake may fluctuate depending on the day of the week. However, little is known about this relationship with physical fitness. 

Considering the different gaps in the literature, the purpose of this study was to assess the effect of diet quality, in a population of different physical fitness, on saliva cortisol while accounting for mood during a peak day of the week and a weekend among men and women. Results from this study will potentially provide a proof-of-concept, that tailored guidelines may be needed to improve mental well-being based on gender, exercise level, diet quality, and time of the week.

## 2. Methods

### 2.1. Study Design

The study protocol was approved by the Institutional Review Board (IRB) at Binghamton University, New York, NY, USA (STUDY 00000143). A modification to collect saliva was approved on 19 February 2019 (MOD00000592). A signed informed consent form was obtained from every participant before the start of the study. This research study was advertised on campus through social media as well as flyers and used a non-probability sampling for subject recruitment. Those interested in participating in the study attended an initial meeting, which was followed by a question-and-answer session. Inclusion criteria were active healthy individuals aged 18 years or older. Exclusion criteria comprised any health condition (cardiovascular, renal, metabolic, neurological, or neuromuscular) that contraindicates exercise or restricts movement. After signing the consent form, participants completed general demographic and health forms. The demographic form included questions about whether the participant is an athlete. If yes, choices were given to select the type of athlete such as Division I, university sports club, or recreational. To further validate the responses, a question on the frequency of exercise was included. Division I and sports clubs were considered student-athletes, as they practice on average over 14 h a week; all others were considered non-athletes.

A total of 48 healthy college students (30 females and 18 males) participated in this experiment. Participants were assigned a study code and were asked to record their dietary intake for three consecutive days using the MyFitnessPal mobile application. Participants also completed the Mood and Anxiety Symptom (MASQ), Kessler Psychological Distress Scale (K10) questionnaires, and the General Nutritional Knowledge Survey (GNKS) on Wednesday and Saturday before the exercise session. The purpose of diet tracking for three days was for a couple of reasons: (1) to identify a potential pattern in dietary intake that could be associated with mood change; and (2) to identify a potential lag time between dietary intake and mood that may explain the potential change in mental status. The purpose of tracking mood on Wednesday and Saturday was to compare a peak weekday versus a downtime. GNKS was included to assess the level of nutritional knowledge among participants and compare it to diet quality. All participants were asked to eat a light breakfast and refrain from eating at least an hour before the exercise session to avoid reactive hypoglycemia during the workout, which may impact cortisol levels. They were also required to rinse their mouth with water prior to saliva provision.

### 2.2. Sample Size Calculation

When conducting a sample size calculation, for a 90% confidence interval and 10% margin of error, wider than the most commonly used values of 95% and 5%, respectively, the minimum sample size was 68, which is higher than our sample size of 48. To address this limitation, a minimum sample size of 41 provides an 80% confidence interval and −/+ 10% margin of error.

### 2.3. Exercise and Saliva Collection

All cycling sessions were carried out in the cycling rooms in the Health and Wellness Studies department at Binghamton University on ergometer stationary bicycles. Four research assistants trained in data and saliva collection oversaw the cycling sessions. Each session comprised, on average, of 10 participants. Upon arrival, participants visited three stations before being cleared for cycling. Station 1 was checking in and making sure that participants had complied with all pre-exercise requirements. Station 2 was for the submission of their dietary logs. Station 3 was for completing all questionnaires. Participants provided the 2 mL of saliva samples at the last station. All specimens were collected in 15 mL bio-reaction tubes. Exercise sessions were conducted between 10 a.m. and 12 p.m. to control for morning cortisol and the circadian rhythm. Since cortisol peaks during early morning hours and gradually decreases, the mid-morning exercise was to compare the baseline cortisol after the peak between SA and NA as well as study the impact of exercise on saliva cortisol. We hypothesized that SA may have higher saliva cortisol throughout the day than NA. The mid-morning was also chosen as energy levels tend to be higher during the earlier hours of the day. All participants started with a 10 min warm-up and 40 min of cycling, at a moderate intensity, which was followed by a 10 min cool-down. The moderate intensity was set at a minimum of 65% of age-adjusted maximum heart rate to avoid skewing the results, as a higher intensity may elevate cortisol levels. At the end of the cycling session and before starting the cool-down, participants provided 2 mL of saliva samples using the same collection technique. Saliva samples that were collected before and after exercise were used to quantify cortisol concentration. Samples were kept on ice and stored at −20 °C. Saliva cortisol was analyzed using saliva cortisol ELISA kits (0.5–100 ng/mL) acquired from Eagle Biosciences (Amherst, NH, USA). Saliva specimens were prepared by centrifugation at 1060× *g* for 15 min, kept at −20 °C for one hour to precipitate the mucin, and then centrifuged once again to remove all particles that may interfere with the antibody binding [13].

### 2.4. Dietary Intake and Cortisol Levels

Once participants signed the consent form, the research assistants generated a MyFitnessPal account for each subject to log in their dietary intake for three days. This mobile application provides a detailed log of nutritional information by breaking down food intake into macro-and micronutrients and displays graphically the relative consumption of each macronutrient based on total calories needed as determined by height, weight, and level of exercise. Logs from MyFitnessPal, macronutrient breakdown, and Alternate Healthy Eating Index (AHEI), as well as a total caloric intake, were extracted for each participant. The AHEI assesses diet quality as guided by the Dietary Guidelines for Americans while being prognostic of chronic disease risk. AHEI scores were calculated using serving size recommendations of the five food groups (whole grains, protein, vegetables, fruit, and dairy) for each participant. Scores ranged from 0 to 50, where a score of 50 characterized a healthy eating pattern. Carbohydrates were further classified into simple and complex carbohydrates to account for their potential effect on cortisol levels. Basal metabolic rate (BMR) was calculated using the Mifflin–St. Jeor equation for each participant [14]. The caloric intake per day (Cal/day) was divided by the calculated BMR to calculate Cal per day/BMR ratios. Participants completed all surveys on Wednesday, as representative of mid-week, and on Saturday, as representative of a weekend day. Survey responses were collected in the morning, at an average time of 11:04 a.m. on Wednesday and 9:27 a.m. on Saturday.

### 2.5. K10, MAQS, and GNKS

K10 has a well-established convergent validity when compared to similar scales [15]. Its criterion validity, as an effective tool for predicting mental health ailments, is considered very good to excellent, with the area under the receiver operating characteristic curve (AUC) ranging from 0.879 with an excellent internal consistency producing a Cronbach alpha of α = 0.93 [16]. K10 consists of a 10-question scale to assess non-specific psychological distress [17]. Questions were based on a five-point Likert scale that ranges from 1 = none of the time to 5 = all of the time. The total score of K10 was used to assess mental distress. Participants also took the Mood and Anxiety Symptoms Questionnaire (MASQ) to distinguish between positive and negative affect or mood. MASQ is based on a tripartite model of anxiety and depression assessment, which distinguishes between several underlying traits of emotional symptoms such as negative affect, anhedonia, low positive affect, and anxious arousal [18,19,20]. MASQ has an excellent reliability with the following Cronbach alpha values: general distress: α = 0.96; anhedonia: α = 0.96; and anxious arousal: α = 0.91 [21]. The MASQ scores also follow a five-point Likert scale that ranges from 1 = no anxious symptoms felt at all to 5 = anxious symptoms felt to the extreme. Similar to the K10 scoring, higher MASQ scores indicate greater negative affect.

### 2.6. Comparison of Nutritional Knowledge between SA and NA

The Nutritional Knowledge Survey’s (GNKS) internal consistency of each section ranges between Cronbach’s α = 0.70–0.97 with a good test-retest reliability of r > 0.7 [21]. GNKS was administered to assess knowledge of nutrition in relation to diet quality. Another aim was to evaluate changes in scores between Wednesday and Saturday to assess any potential interest in improving nutritional knowledge. The hypothesis was that athletes will have a higher score on Saturday than non-athletes. In this study, student-athletes (SA) are defined as Division I and sports club athletes with similar levels of practice and competition.

### 2.7. Statistical Analysis

Since the sample size was relatively small, few suggestions were adapted from Hopkin et al. [22], such as focusing on the variables with the highest yield and testing interactions between variables. The Wilcoxon rank-sum test compared SA and NA concerning different variables, and the Wilcoxon signed-rank test evaluated individuals within each group (SA or NA) before and after exercise and on Wednesday and Saturday. In addition, the Pearson chi-square test with Yates correction for continuity was used to compare the percentage of females among SA and NA. To explore the relationship between different variables such as nutritional factors, cortisol level, mood, and mental distress, a linear regression analysis was employed. The significance level was *p* < 0.1 since the study compared several factors for a limited number of participants. Data were analyzed in R, v3.5.0. [23].

## 3. Results

Our data set includes responses from 48 college students (23 athletes versus 25 non-athletes). Since there were several variables of interest, the first step was to explore the data at hand. Table 1 summarizes the findings for SA and NA and compares the two groups in terms of different factors. The reported BMI levels have a range of 17.5 to 34.5. Three individuals (all women, one athlete) have BMI below the healthy range (18.5–25), and eighteen individuals (8 women, 7 athletes) have BMI levels above the healthy range.

### 3.1. Characteristics of SA and NA

**Figure 1:**
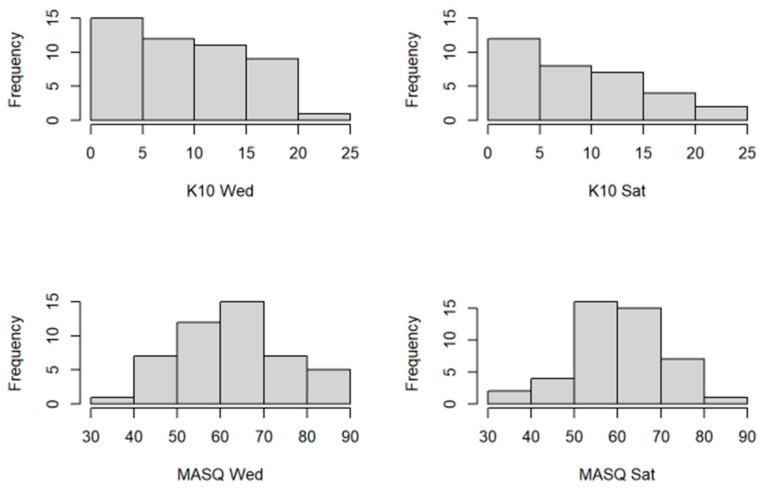
Histogram of the K10 and MASQ scores on Wednesday vs. Saturday, reflecting the distribution of mental well-being scores among students.

Figure 1 illustrates the distribution of mental well-being scores among students. Based on the results shown in Table 1, there is no statistically significant difference between cortisol levels of SA and NA before exercising. However, for NA, salivary cortisol levels significantly dropped post-exercise, leading to a significant difference between SA and NA. In other words, SA maintained a higher cortisol level after exercise. Additionally, there is no statistically significant difference between MASQ Wednesday scores of SA and NA. However, SA MASQ Saturday scores significantly improved, leading to a significant difference in the MASQ Saturday scores of SA and NA. Accordingly, these results suggest that those with higher physical fitness levels experienced better control over their emotional arousal during downtime. It is interesting to note that although SA have significantly higher cortisol levels on Saturday, they have significantly better MASQ Saturday scores. There is no statistically significant difference between the K10 score of SA and NA, for both Wednesday and Saturday, which suggests that individuals maintain a comparable level of mental status, regardless of fitness level. There is no statistically significant difference between the nutritional knowledge of SA and NA, but for NA, surprisingly, their knowledge score significantly increased on Saturday, which suggests that NA researched the nutritional questions and that SA may assume to have the basic knowledge of major nutrition facts. Among other factors listed in Table 1, SA have significantly higher calories per BMR, higher consumption of simple carbohydrates and fat, and lower consumption of vegetables. SA are also significantly younger than NA. There was no significant difference in AHEIs or food groups between SA and NA. Since there were differences in MASQ and K10 scores on Wednesday and Saturday, there was a need to further evaluate these parameters using regression models. 

### 3.2. Relationship between MASQ and K10 Scores

In the first linear regression model, MASQ Wednesday scores were considered the dependent variable, and the K10 Wednesday scores as the only independent variable (Table 2). On Wednesday, there was a very significant positive association between K10 and MASQ scores (adjusted R-squared is 0.14).

Next, the same model was repeated for Saturday. The significant positive association between K10 and MASQ scores depicted on Wednesday dissipated on Saturday (Table 3).

To effectively study the association between MASQ, K10 and cortisol levels, a Pearson’s correlation coefficient test was used (Table 4). There are strong positive correlations between K10 scores (0.63) (Wednesday and Saturday), and between MASQ scores (0.68) (Wednesday and Saturday). There is also a considerable positive correlation between K10 Wednesday and MASQ Wednesday scores (0.40), but this correlation became non-significant between MASQ Saturday and K10 Saturday. An interesting observation noted here is that cortisol level before and after exercise, without considering other variables, does not have a strong correlation with MASQ or K10 on either day (Table 4), which suggests that cortisol is not the only determinant of mental status, but its effect is contingent on other factors. 

### 3.3. MASQ Wednesday, K10 Wednesday, and Cortisol before Exercise

To better understand the relationship between negative affect, mental distress, cortisol and exercise, and time of the week, a linear regression analysis was used to simulate these relationships. In this model, MASQ scores were the dependent variable, while K10 scores and cortisol levels (before exercise) were the independent variables. When cortisol levels were factored in, a strong significant positive relationship between MASQ Wednesday and K10 Wednesday scores emerged, suggesting that negative affect and mental distress may be associated during a peak day of the week. In addition, a significant negative relationship between MASQ Wednesday and cortisol levels before exercise surfaced (adjusted R-squared is 0.1985), proposing that lower cortisol values before exercise are associated with a better mood during a peak day of the week (Table 5).

### 3.4. MASQ Wednesday, K10 Wednesday, and Cortisol after Exercise

The same analysis was repeated for cortisol after exercise. Interestingly, there is no significant association between cortisol levels after exercise and K10 Wednesday scores (Table 6), suggesting that cortisol levels after exercise may not be an accurate indicator of mental status. 

### 3.5. MASQ Saturday, K10 Saturday, and Cortisol before Exercise

As with the previous models, the MASQ Saturday score was considered as the dependent variable, and the K10 Saturday score and cortisol levels before exercising as the independent variables. A significant positive relationship between MASQ Saturday and K10 Saturday scores was detected when cortisol levels were included, but there was no statistically significant relationship with cortisol before exercise. This means that for the same cortisol value before exercise, individuals with higher K10 Saturday scores tend to have higher MASQ Saturday scores (Table 7) and that cortisol level is not associated with mental status, which confirmed the previous observation. 

### 3.6. MASQ Saturday, K10 Saturday, and Cortisol after Exercise

The preceding analysis was repeated with the inclusion of cortisol after exercise as an independent variable. The association between MASQ Saturday and K10 Saturday vanished when cortisol after exercise was considered. The absence of significance depicted for MASQ Wednesday and cortisol after exercise resurfaced for MASQ Saturday scores (Table 8), confirming the findings from previous models. 

Taken together, the relationship between K10, MASQ, and cortisol levels before exercise is stronger on Wednesday compared with Saturday. In other words, higher cortisol levels before exercise correlated with higher levels of negative affect and mental distress on a peak day of the week. However, exercise seems to reduce this close association. The next step was to investigate the relationship between MASQ, K10, cortisol, dietary factors, being an athlete (or physically fit), sex, BMI, and age using different regression models to comprehend the integration of these factors.

### 3.7. Model One 

In this model, the relationship between the change in cortisol level after exercise with other variables of interest was assessed. Change in cortisol is defined as the cortisol level before exercise minus the cortisol level after exercise. Cortisol difference is the dependent variable, while sex, age, BMI, being an athlete, calories per BMR, and consumption of sugar, protein, fat, simple carbohydrates, and complex carbohydrates are considered independent variables. SA and NA showed differences in changes in their cortisol levels after exercise. The results suggest that higher BMI values and lower fat consumption increase cortisol difference or a higher reduction in cortisol level after exercise. Since SA’s diet includes significantly more fat (247.9 ± 88.2 versus 174.2 ± 73.9 with *p*-value < 0.004), the analysis was repeated to differentiate between SA and NA (Table 9).

The repeated analysis revealed that a higher BMI and a lower-fat diet are significant contributors to the cortisol difference in SA (Table 10). There are no significant variables found when the analysis was repeated for NA, which suggests that changes in cortisol levels may be related to higher workout intensity due to advanced physical fitness. 

### 3.8. Model Two 

In this model, the aim was to study the factors that influenced K10 Saturday scores. Therefore, K10 Saturday scores were the dependent variable, and sex, age, BMI, being an athlete, calories per BMR, cortisol level before exercise, MASQ score on Saturday, K10 score on Wednesday, and consumption of sugar, protein, fat, simple carbohydrates, and complex carbohydrates were the independent variables. On average, females have lower K10 Saturday scores, which suggests that women may benefit from downtime to improve mental well-being. Lower protein and simple carbohydrate consumption and higher K10 Wednesday scores were associated with an increase in K10 Saturday scores (adjusted R-squared is 0.4379) (Table 11). 

### 3.9. Model Three

The purpose of this analysis was to assess the factors that influenced MASQ Saturday scores (adjusted R-squared is 0.5926). This model considered MASQ Saturday scores as the dependent variable, while sex, age, BMI, being an athlete, calories per BMR, cortisol level before exercise, K10 score on Saturday, MASQ score on Wednesday, and consumption of sugar, proteins, fat, simple carbohydrates, and complex carbohydrates are considered independent variables. Athletes have significantly lower MASQ Saturday scores, after adjusting for all other factors. Moreover, there is a positive association between MASQ Saturday, MASQ Wednesday, and cortisol levels before exercise. Furthermore, lower consumption of fat and higher consumption of proteins seem to improve MASQ Saturday scores (Table 12). 

### 3.10. Model Four

This analysis was carried out to identify the dietary factors that may have led to the MASQ Saturday. Therefore, the previous analysis was repeated with the inclusions of food groups. Hence, we considered MASQ Saturday scores the dependent variable, and sex, age, BMI, being an athlete, calories per BMR, cortisol level before exercise, MASQ Wednesday scores, and consumption of refined grain, whole grain, fruit, vegetables, dairy, meat, eggs, nuts, beans, and caffeine as independent variables. Cortisol before exercise and egg consumption was negatively associated with MASQ Saturday scores, while beans revealed a positive relationship. However, since we included more variables in this model with small sample size, we take these results with caution. An analysis of cortisol difference and the K10 Saturday model did not return any significant results, potentially due to a high correlation between independent variables and/or due to the small sample size (Table 13). 

## 4. Discussion

The purpose of the study was to investigate the relationship between dietary factors, physical fitness, and salivary cortisol levels (before and after exercise), and their effect on mood while considering the time of the week. The rationale of the study is to decipher the different factors that may impact mood in populations of different fitness levels and time settings while accounting for dietary factors and cortisol levels. To our knowledge, this is the first study that looked at these integrated variables.

The study confirms previously reported findings and fills several gaps in the literature. The main findings of this research include: (1) There is a potential robust link between dietary factors, cortisol levels, mood, and time of the week. This suggests that customization of diet and lifestyle factors based on time of the week and fitness level may improve mood. The link between saliva cortisol and time of the week was previously addressed. Kim et al. [24] reported that saliva cortisol obtained 30 min after awakening produced similar levels on workdays but were significantly different from the saliva cortisol levels on Sunday, which agrees with our findings. In addition, saliva cortisol levels may not be directly linked to negative affect or mental well-being but to an assortment of factors when mental distress exists. This finding suggests that when assessing cortisol levels and mood it is necessary to evaluate the confounding factors. This notion was implied by Melin et al. [25] who reported that higher cortisol levels in type I diabetics with depression were associated with a myriad of lifestyle factors such as smoking, inactivity, and season. In fact, several other factors were reported to be associated with cortisol levels such as age, hormone therapy, alcohol use, and genetic as well as personality factors, just to name a few [26]. It is also important to note that modulation of these modifiable factors may improve cortisol levels; (2) During a peak weekday, individuals who experience negative affect are more likely to experience mental distress, but the likelihood of these emotions remaining high subsides during downtime such as a weekend. In addition, physical fitness may play a role in improving mood during downtime; (3) Cortisol levels after exercise may not be a good indicator of mental status, but a gage of workout intensity; (4) Physically fit individuals may have a false sense of nutritional knowledge, which may be impacting the quality of their diet. 

### 4.1. Differences in Cortisol Levels between SA and NA

Contrary to our expectation, SA did not have significantly higher cortisol levels at baseline. One of the interesting findings was cortisol levels after exercise was different between SA and NA. The latter experienced a drop in their cortisol level post-workout, while SA maintained fairly the same level. The fact that both groups had no statistical significance in their cortisol level before exercise suggests that the changes recorded in cortisol values were exercise-related. One potential explanation for the differential cortisol level is the intensity of exercise. Athletes are more likely to exercise at a higher intensity than non-athletes. Cortisol concentrations were reported to be positively associated with speed, power, and strength in professional athletes [27]. Siart et al. reported that athletes tend to experience elevated cortisol before a competition to promote arousal and suppress pain [28]. In addition, low to moderate exercise has been linked to a reduction in circulating cortisol levels [29], which may explain the differential findings. Since participants were asked to exercise at a moderate intensity, it is plausible that SA exercised at a higher intensity than NA. Another potential explanation is diet quality. Although no significant differences in AHEI scores were detected between both groups, SA tends to consume a high-fat simple carbohydrate diet and more calories per BMR, which may increase inflammation and cortisol secretion [30,31]. High glycemic food (such as simple carbohydrates and refined grains) leads to blood sugar fluctuation, which has been associated with changes in mood and a higher risk for mental distress [32]. However, optimal protein to carbohydrate intake may offset this fluctuation. Since protein intake was not significantly different between SA and NA, this hints that SA had a lower ratio of proteins to carbohydrates. Mechanistically, high-quality proteins provide ample tryptophan (Trp), the precursor of serotonin, and decrease the glycemic load of carbohydrates. Trp is transported to the brain by an insulin-dependent system [33]; therefore, rapid changes in blood glucose due to high glycemic food intake produce oscillations in serotonin levels, which impacts mood [34].

### 4.2. Nutritional Knowledge

Another fascinating observation was that being athletic may give a false sense of nutritional knowledge. There was no statistically significant difference between nutritional knowledge of SA and NA on Wednesday. However, for the retest on Saturday, NA knowledge scores significantly increased, suggestive of potential research performed by NA on the undetermined nutritional questions. A survey assessing nutritional knowledge of student-athletes (personal communication), conducted by one of the principal investigators, suggested that the major sources of nutritional information for SA are coaches, the web, and parents. A recent report assessing nutritional knowledge of Division I athletes and their coaches reported 80% vs. 54%, respectively, of misinformation [35]. Another study that assessed nutritional knowledge in athletic trainers, collegiate athletes, and coaches reported the lowest knowledge in athletes followed by coaches, while athletic trainers scored higher on nutritional knowledge [36]. The fact that SA nutritional knowledge scores did not improve on Saturday hints toward an underlying confidence in their nutritional knowledge. It is worth mentioning that, based on the dietary logs, the diet of SA is considered imbalanced. Therefore, there is a need to include formal nutrition education as part of the SA coursework or training schedule.

### 4.3. MASQ versus K10 Scores

Since MASQ and K10 scores did not produce similar responses as anticipated, we wished to study their performances between Wednesday and Saturday, individually and combined, and independently of all factors. There was no statistically significant difference at baseline between MASQ Wednesday scores of SA and NA. However, SA had an improved MASQ Saturday score, while K10 scores did not reveal any significant differences. In addition, there is a considerable positive correlation between K10 Wednesday and MASQ Wednesday scores, but this correlation became non-significant on Saturday. These findings propose that negative affect and mental distress may happen together on a peak day of the week. Stone et al. [37] reported better moods on Friday and weekends compared with weekdays. Similar results were reported by Ryan et al. [38] who described that mood variability during weekdays is highly associated with the type of work, and that this pattern was observed in both men and women. Since MASQ assesses mood and negative affect, changes in daily circumstances may have impacted these scores [39]. It is noteworthy to mention that although SA had higher cortisol levels after exercise, they produced lower MASQ Saturday scores, which proposes that those with higher physical fitness experience a faster improvement in mood during downtime.

Subsequently, there was a need to capture the relationship between MASQ and K10 scores on Wednesday and Saturday independently of other factors. The correlational study showed a very strong positive association between K10 scores (Wednesday and Saturday) and MASQ scores (Wednesday and Saturday) (*p* < 0.0001) and a lesser significant association between MASQ and K10 scores (Wednesday and Saturday) (*p* < 0.05). This finding confirmed the notion that negative affect and mental distress may happen together on a peak day of the week, which may be explained by the type of work or tasks at hand [38] and that distress may likely linger during downtime. 

### 4.4. MASQ, K10, and Cortisol Levels

Another interesting observation noted here is that cortisol level before and after exercise, without considering the confounding factors, does not correlate with either MASQ or K10. To our knowledge, we are the first to report this concept. However, when other variables were measured, cortisol before exercise showed some significant associations and promoted a significant relationship between MASQ and K10 scores. This is in accordance with previous reports that suggested that lifestyle factors may be impacting cortisol levels independently of mood [25,26]. For instance, K10 Wed scores and MASQ Wed scores, as well as MASQ Sat and K10, Sat scores were correlated when cortisol before exercise was included as an independent variable. Interestingly, this significant association died out between MASQ and K10 scores when cortisol after exercise was incorporated into the model. Since cortisol levels were not assessed on Wednesday but Saturday (and during a downtime), this suggests a potential lag-time between negative affect and cortisol levels [40]. Taken together, it seems that the relationship between K10, MASQ, and cortisol levels before exercise is stronger on a peak day (Wednesday) compared with a weekend (Saturday). This finding proposes a significant role for exercise in improving mood, especially during downtime.

### 4.5. Gender, MASQ, K10, BMI, Cortisol Levels, Food Groups, and BMI

The next step was to investigate the relationship between the MASQ, K10, cortisol, dietary factors, being an athlete (or physically fit), sex, BMI, and age using different regression models

#### 4.5.1. Cortisol Levels and BMI

Cortisol difference between pre-and post-exercise was associated with higher BMI values and lower fat consumption. Nevertheless, when we compared this finding between SA and NA, only SA showed this association. Therefore, SA with higher normal BMI and consuming less fat experienced a decline in cortisol that was not experienced by SA with lower BMI values and higher fat consumption. It is worth noting that the cohort’s BMI fell within normal ranges. Therefore, higher BMI in our sample refers to higher values within the normal range (18.5–24.99 kg/m^2^). However, BMI of ≥25 kg/m^2^ was also reported to be associated with lower basal cortisol [41], with a potential U-shaped curve effect [42]. This may be explained by the bioenergetic characteristics of fat used as a source of energy during exercise. Low body fat may stimulate cortisol and adrenaline release to promote glycogenolysis and gluconeogenesis to preserve the limited fat stores. Meanwhile, high body fat may be associated with some level of insulin resistance that may trigger the stress response due to a lack of efficient glucose uptake by muscle cells [43].

#### 4.5.2. K10 Scores

The subsequent step was to examine the factors that affected the changes in K10 scores between the middle (Wednesday) versus the end of the week (Saturday). On average, females produced lower K10 scores on Saturday, and NA had lower K10 scores in general. The former finding was a surprise as women have a higher risk for mental distress when compared with men. However, this finding suggests that women may have a better mental status during downtime or, in other words, women may benefit from taking time off. This also suggests that when assessing women’s mood, it is essential to consider the time of the week for accurate results. 

Higher calories per BMR and K10 Wednesday scores were associated with an increase in K10 Saturday scores. However, higher consumption of protein and simple carbohydrates were linked to a decrease in the K10 Saturday score. This finding confirms the theory stated above about an optimal ratio for protein to simple carbohydrates that are typically coupled with stable blood sugar and enhanced neurotransmitter synthesis [44].

#### 4.5.3. MASQ Scores

The same analysis was carried out to assess the factors that influenced MASQ Saturday scores. Athletes have significantly lower MASQ Saturday scores, after adjusting for all other factors. Additionally, a positive association between MASQ Saturday, MASQ Wednesday, and cortisol levels before exercise was detected. Likewise, lower consumption of fat and higher consumption of proteins seem to improve the MASQ Sat score. 

Combining the findings from MASQ and K10 models, the association between MASQ and cortisol before exercise, when all variables were included, remained strong. Conversely, this association became non-significant between cortisol before exercise and K10 scores. This suggests that cortisol levels are potentially more sensitive to negative affect (short-term) than mental distress (long-term) when considering dietary and lifestyle factors. Herane-Vives et al. reported lower cortisol levels in atypical depressed individuals, proposing a role for environmental factors for this decrease [45]. This finding recommends that dietary adjustments may improve cortisol levels and short-term affect, which may eventually improve mental well-being. 

#### 4.5.4. The Impact of the Diet on Mood

The changes in MASQ score between Wednesday and Saturday were assessed in relation to all nutritional factors (instead of nutritional categories). The results show that egg consumption was negatively associated with MASQ Saturday scores, while beans revealed a positive relationship. Although these results are taken with caution due to the small sample size in relation to the number of variables in the model, there is scientific evidence that supports this association. Eggs are high in tryptophan and choline. The latter is required to produce acetylcholine, which is a major neurotransmitter in the nervous system. In a large population-based study, plasma choline concentrations were negatively correlated with anxiety, but not with depression [46], which may also explain why eggs were not associated with K10 scores. High consumption of beans is typically related to a low animal protein intake. Some evidence has linked the vegetarian diet to an increased risk for mental distress [47]. In addition, good protein quality was associated with mental well-being in young adults, which may indirectly support this finding as well [48]. 

#### 4.5.5. Strengths and limitations of the study

The study fills different gaps in the literature and provides evidence of new potential associations between cortisol levels and mood as a proof-of-concept that could be used for future research. For instance, since women are known to have higher levels of mental distress, this study suggests the need to factor in the time of the week to accurately assess their mental status. In addition, the study used robust analytical methods to extract the findings. However, the limitation of the study is the sample size which may affect the accuracy and interpretability of our results. For this sample size, the confidence interval is slightly over 80% with the corresponding margin of error. Few actions were taken to maximize the power of the study, as suggested by Hopkin et al. [22]. Another action taken was to use a larger alpha value (*p* < 0.1) to account for both the probabilities of both type I and type II errors. In addition, self-reporting of dietary intake may have included distorted portion size, the incorrect food type, or the preparation method that may have altered the nutritional breakdown of nutrients. Finally, several findings are noted as a proof-of-concept that will require further investigation. 

## 5. Conclusions and Future Direction

This study fills several gaps in the literature and suggests a further direction. Our results propose that a peak day of the week may be associated with different mental stressors, but those who display higher physical fitness may relax faster during downtime. Women are more likely to improve their mental well-being by taking some time off. Another interesting observation is cortisol levels are not tied directly to mental status but are modulated by dietary and lifestyle factors. This is an important concept as these factors are modifiable in nature. Due to the sample size of this study, there is a need for larger controlled studies to confirm these findings. As for future directions, since athletes’ diets are high in fat, it would be interesting to assess if any specific type of fat may be directly linked to cortisol levels. Another interesting hypothesis to test is that consuming a high-quality diet, including healthy fats such as monounsaturated fat and a healthy ratio of omega 6/omega 3 fat, by athletes improves K10 scores.

## Figures and Tables

**Table 1:** Summary of characteristics of student-athletes and non-athletes. * *p* < 0.1.

Variables	Student-Athletes (*n* = 23)	Non-Athletes (*n* = 25)	*p*-Value
Saliva Cortisol Before	5.46 ± 6.54	5.71 ± 6.26	0.92
Saliva Cortisol After	5.80 ± 7.38	3.19 ± 5.16	* 0.02
***p*-Value (Before and After)**	**0.80**	* 0.002	-
MASQ Wed	61.30 ± 12.58	64.04 ± 10.90	0.39
MASQ Sat	56.33 ± 8.70	62.00 ± 10.19	* 0.04
***p*-Value (Wed and Sat)**	* 0.02	**0.16**	**-**
K10 Score Wed	8.73 ± 4.69	10.64 ± 7.37	0.38
K10 Score Sat	8.73 ± 4.85	9.72 ± 7.35	1
***p*-Value (Wed and Sat)**	**0.72**	**0.12**	**-**
Knowledge Score Wed	9.50 ± 2.94	7.96 ± 4.02	0.34
Knowledge Score Sat	9.93 ± 2.49	9.67 ± 2.54	0.62
***p*-Value (Wed and Sat)**	**0.28**	* 0.03	-
AHEI	38.40 ± 7.05	37.82 ± 8.24	0.62
Calories per BMR	1.28 ± 0.41	1.04 ± 0.34	* 0.049
BMI	24.79 ± 3.92	24.55 ± 4.01	0.84
Female	16 (69.6%)	14 (56%)	0.82
Age	21.09 ± 2.83	23.4 ± 3.14	* 8.7 × 10^−5^
Simple Carbs	545.68 ± 227.52	384.59 ± 143.23	* 0.03
Complex Carbs	158.95 ± 134.121	144.77 ± 104.72	0.79
Proteins	267.11 ± 124.18	233.56 ± 116.57	0.38
Fat	247.98 ± 88.24	174.22 ± 73.88	* 0.004
Refined Grains	8.70 ± 6.72	6.90 ± 3.86	0.55
Whole Grains	1.60 ± 1.14	3.64 ± 3.96	0.41
Fruits	3.90 ± 3.68	2.61 ± 2.73	0.33
Vegetables	1.90 ± 1.78	3.82 ± 2.05	* 0.08
Dairy	4.00 ± 2.03	5.03 ± 5.26	0.91
Lean Meat	4.60 ± 3.80	4.53 ± 4.22	1
Eggs	1.20 ± 2.17	2.11 ± 3.05	0.48
Nuts	0.80 ± 1.30	0.83 ± 1.49	1
Alcohol	1.50 ± 3.35	1.58 ± 4.70	1
Caffeine	1.80 ± 1.92	1.47 ± 2.01	0.59
Beans	0 ± 0	0.56 ± 1.30	0.16

**Table 2:** Regression result of MASQ Wednesday scores (dependent variable) and K10 Wednesday scores (independent variable). * *p* < 0.1.

Independent Variable	Coefficient	Standard Error	*p*-Value
Intercept	55.67	2.95	* 0.000
K10 Score Wed	0.75	0.25	* 0.005

**Table 3:** Regression results of MASQ Saturday scores (dependent variable) and K10 Saturday scores (independent variable). * *p* < 0.1.

Independent Variable	Coefficient	Standard Error	*p*-Value
Intercept	53.98	3.30	* 0.000
K10 Sat	0.50	0.30	* 0.1

**Table 4:** Pearson’s correlation coefficient test results for K10 and MASQ scores on Wednesday and Saturday and saliva cortisol levels before and after exercise.

	K10 Wed	MASQ Wed	K10 Sat	MASQ Sat	Cortisol Before	Cortisol After
K10 Wed	1	0.40 *	0.63 **	0.38 *	0.10	−0.11
MASQ Wed	-	1	0.26	0.68 **	−0.26	−0.15
K10 Sat	-	-	1	0.29	0.20	0.03
MASQ Sat	-	-	-	1	−0.005	−0.12
Cortisol Before	-	-	-	-	1	0.13
Cortisol After	-	-	-	-	-	1

* *p* < 0.05; ** *p* < 0.0001.

**Table 5:** Regression results of MASQ Wednesday scores (dependent variable), K10 Wednesday score, and saliva cortisol level before exercise (independent variables). * *p* < 0.1.

Independent Variable	Coefficient	Standard Error	*p*-Value
Intercept	58.0	3.07	* 0.000
K10 Score Wed	0.8	0.25	* 0.002
Cortisol Before	−0.5	0.24	* 0.047

**Table 6:** Regression results of MASQ Wednesday scores (dependent variable), K10 Wednesday scores, and saliva cortisol levels after exercise (independent variables). * *p* < 0.1.

	Coefficient	Standard Error	*p*-Value
Intercept	56.65	3.27	* 0.000
K10 Score Wed	0.73	0.26	* 0.007
Cortisol After	−0.18	0.25	0.48

**Table 7:** Regression results of MASQ Saturday scores (dependent variable), K10 Saturday scores, and saliva cortisol levels before exercise (independent variables). * p < 0.1.

Independent Variable	Coefficient	Standard Error	*p*-Value
Intercept	54.42	3.56	* 0.000
K10 Sat	0.5	0.31	* 0.09
Cortisol Before	−0.1	0.27	0.72

**Table 8:** Regression result of MASQ Saturday scores as the dependent variable and K10 Saturday scores and saliva cortisol levels after exercise (independent variables). * *p* < 0.1.

Independent Variable	Coefficient	Standard Error	*p*-Value
Intercept	54.96	3.55	* 0.000
K10 Score Sat	0.50	0.30	0.1
Cortisol After	−0.20	0.25	0.45

**Table 9:** Regression results of cortisol change after exercise (dependent variable) and sex, age, BMI, athlete, calories per BMR, sugar, proteins, fat, simple carbohydrates, and complex carbohydrates (independent variables). * *p* < 0.1.

Independent Variable	Coefficient	Standard Error	*p*-Value
Intercept	−12.67	15.86	0.43
Sex	−1.48	3.55	0.68
Age	−0.06	0.53	0.91
BMI	0.82	0.39	* 0.04
Athlete	1.62	3.12	0.61
Calories per BMR	13.11	7.80	0.10
Sugar	0.01	0.02	0.65
Proteins	0.005	0.02	0.82
Fat	−0.07	0.03	* 0.03
Simple Carbohydrates	−0.02	0.017	0.33
Complex Carbohydrates	−0.01	0.017	0.59

**Table 10:** Significant factors in the regression analysis of cortisol change after exercise in athletes (dependent variable) and sex, age, BMI, athlete, calories per BMR, sugar, proteins, fat, simple carbohydrates, and complex carbohydrates (independent variables) (adjusted R-squared 0.13). * *p* < 0.1.

Independent Variable	Coefficient	Standard Error	*p*-Value
BMI	1.27	0.58	* 0.05
Fat	−0.11	0.05	* 0.04

**Table 11:** Regression result of K10 Saturday scores (dependent variable) and sex, age, BMI, athlete, calories per BMR, cortisol level before exercise, MASQ scores on Saturday, K10 scores on Wednesday, sugar, proteins, fat, simple carbohydrates, and complex carbohydrates (independent variables). * *p* < 0.1.

Independent Variable	Coefficient	Standard Error	*p*-Value
Intercept	24.67	18.52	0.20
Sex	−10.59	4.10	* 0.018
Athlete	3.85	3.65	0.31
Age	−0.61	0.66	0.37
BMI	−0.03	0.28	0.92
Calories per BMR	11.1	7.36	0.15
Sugar	0.04	0.03	0.15
Proteins	−0.04	0.022	* 0.083
Fat	0.0005	0.02	0.98
Simple Carbohydrates	−0.03	0.016	* 0.09
Complex Carbohydrates	−0.01	0.015	0.43
K10 Wed	0.57	0.25	* 0.03
Cortisol Before	0.22	0.15	0.17
MASQ Sat	0.03	0.12	0.83

**Table 12:** Regression results of MASQ Saturday score (dependent variable) and sex, age, BMI, athlete, calories per BMR, cortisol levels before exercise, K10 scores on Saturday, MASQ scores on Wednesday, sugar, proteins, fat, simple carbohydrates, and complex carbohydrates (independent variables). * *p* < 0.1.

Independent Variable	Coefficient	Standard Error	*p*-Value
Intercept	27.05	22.92	0.25
Sex	−1.05	6.40	0.87
Athlete	−11.68	3.71	* 0.005
Age	0.44	0.78	0.58
BMI	−0.55	0.38	0.16
Calories per BMR	−3.11	10.83	0.78
Sugar	−0.02	0.03	0.41
Proteins	−0.06	0.03	* 0.099
Fat	0.06	0.03	* 0.090
Simple Carbohydrates	0.03	0.02	0.16
Complex Carbohydrates	0.03	0.02	0.18
MASQ Wed	0.51	0.13	* 0.0008
K10 Sat	−0.23	0.03	0.46
Cortisol Before	0.67	0.23	* 0.009

**Table 13:** Regression results of MASQ Saturday scores (dependent variable) and sex, age, BMI, athlete, calories per BMR, cortisol level before exercise, MASQ scores on Wednesday, refined grain, whole grain, fruit, vegetables, dairy, meat, eggs, nuts, beans, and caffeine (independent variable). * *p* < 0.1.

Independent Variable	Coefficient	Standard Error	*p*-Value
Intercept	68.89	34.47	0.12
Sex	−0.95	4.12	0.83
Athlete	12.96	6.97	0.14
Age	0.52	0.60	0.44
BMI	−2.16	0.70	* 0.09
Calories per BMR	−15.59	4.11	* 0.06
Refined Grain	1.44	0.78	0.14
Whole Grain	1.15	0.98	0.31
Fruit	−2.46	1.18	0.11
Vegetables	−0.26	0.86	0.78
Dairy	0.33	0.67	0.64
Lean Meat	0.52	0.53	0.38
Eggs	−3.83	1.13	* 0.08
Nut	−2.04	2.01	0.37
Beans	11.5	2.3	* 0.04
Caffeine	−0.72	1.52	0.66
MASQ Wed	0.27	0.23	0.31
Cortisol Before	0.10	0.69	0.89

## Data Availability

Data will be provided upon reasonable request.

## References

[1] Silverman M.N., Deuster P.A. (2014). Biological mechanisms underlying the role of physical fitness in health and resilience. Interface Focus.

[2] Abrantes A.M., Strong D.R., Cohn A., Cameron A.Y., Greenberg B.D., Mancebo M.C., Brown R.A. (2009). Acute changes in obsessions and compulsions following moderate-intensity aerobic exercise among patients with obsessive-compulsive disorder. J. Anxiety Disord..

[3] Craft L.L., Perna F.M. (2004). The Benefits of Exercise for the Clinically Depressed. Prim. Care Companion J. Clin. Psychiatry.

[4] Strickland J.C., Smith M.A. (2014). The anxiolytic effects of resistance exercise. Front. Psychol..

[5] Viboolvorakul S., Patumraj S. (2014). Exercise training could improve age-related changes in cerebral blood flow and capillary vascularity through the upregulation of VEGF and eNOS. BioMed Res. Int..

[6] Bromberg-Martin E.S., Matsumoto M., Hikosaka O. (2010). Dopamine in motivational control: Rewarding, aversive, and alerting. Neuron.

[7] Heisler L.K., Zhou L., Bajwa P., Hsu J., Tecott L.H. (2007). Serotonin 5-HT(2C) receptors regulate anxiety-like behavior. Genes Brain Behav..

[8] Begdache L., Chaar M., Sabounchi N., Kianmehr H. (2019). Assessment of dietary factors, dietary practices and exercise on mental distress in young adults versus matured adults: A cross-sectional study. Nutr. Neurosci..

[9] Kadmiel M., Cidlowski J.A. (2013). Glucocorticoid receptor signaling in health and disease. Trends Pharmacol. Sci..

[10] Stults-Kolehmainen M.A., Sinha R. (2014). The effects of stress on physical activity and exercise. Sports Med. Auckl. N.Z..

[11] Strasser B., Fuchs D. (2015). Role of physical activity and diet on mood, behavior, and cognition. Neurol. Psychiatry Brain Res..

[12] Pearlmutter P., DeRose G., Samson C., Linehan N., Cen Y., Begdache L., Won D., Koh A. (2020). Sweat and saliva cortisol response to stress and nutrition factors. Sci. Rep..

[13] Staufenbiel S.M., Penninx B.W.J.H., Spijker A.T., Elzinga B.M., van Rossum E.F.C. (2013). Hair cortisol, stress exposure, and mental health in humans: A systematic review. Psychoneuroendocrinology.

[14] Frankenfield D., Roth-Yousey L., Compher C. (2005). Comparison of Predictive Equations for Resting Metabolic Rate in Healthy Nonobese and Obese Adults: A Systematic Review. J. Am. Diet. Assoc..

[15] Cornelius B.L.R., Groothoff J.W., Van Der Klink J.J.L., Brouwer S. (2013). The performance of the K10, K6 and GHQ-12 to screen for present state DSM-IV disorders among disability claimants. BMC Public Heal..

[16] Kessler R.C., Andrews G., Colpe L.J., Hiripi E., Mroczek D.K., Normand S.L.T., Walters E.E., Zaslavsky A.M. (2002). Short screening scales to monitor population prevalences and trends in non-specific psychological distress. Psychol. Med..

[17] Andrews G., Slade T. (2001). Interpreting scores on the Kessler Psychological Distress Scale (K10). Aust. N. Z. J. Public Heal..

[18] Clark L.A., Watson D. (1991). Tripartite model of anxiety and depression: Psychometric evidence and taxonomic implications. J. Abnorm Psychol.

[19] Wardenaar K.J., van Veen T., Giltay E.J., de Beurs E., Penninx B.W., Zitman F.G. (2010). Development and validation of a 30-item short adaptation of the Mood and Anxiety Symptoms Questionnaire (MASQ). Psychiatry Res..

[20] De Beurs E., den Hollander-Gijsman M.E., Helmich S., Zitman F.G. (2007). The tripartite model for assessing symptoms of anxiety and depression: Psychometrics of the Dutch version of the mood and anxiety symptoms questionnaire. Behav. Res. Ther..

[21] Parmenter K., Wardle J. (1999). Development of a general nutrition knowledge questionnaire for adults. Eur. J. Clin. Nutr..

[22] Hopkin C.R., Hoyle R.H., Gottfredson N.C. (2015). Maximizing the Yield of Small Samples in Prevention Research: A Review of General Strategies and Best Practices. Prev. Sci. Off. J. Soc. Prev. Res..

[23] R Core Team (2020). R: A Language and Environment for Statistical Computing.

[24] Kim M.-S., Lee Y.-J., Ahn R.-S. (2010). Day-to-day differences in cortisol levels and molar cortisol-to-DHEA ratios among working individuals. Yonsei Med. J..

[25] Melin E.O., Thunander M., Landin-Olsson M., Hillman M., Thulesius H.O. (2014). Depression, smoking, physical inactivity and season independently associated with midnight salivary cortisol in type 1 diabetes. BMC Endocr. Disord..

[26] Kudielka B.M., Hellhammer D.H., Wüst S. (2009). Why do we respond so differently? Reviewing determinants of human salivary cortisol responses to challenge. Psychoneuroendocrinology.

[27] Crewther B.T., Lowe T., Weatherby R.P., Gill N., Keogh J. (2009). Neuromuscular performance of elite rugby union players and relationships with salivary hormones. J. Strength Cond. Res..

[28] Siart B., Nimmerichter A., Vidotto C., Wallner B. (2017). Status, Stress and Performance in Track and Field Athletes during the European Games in Baku (Azerbaijan). Sci. Rep..

[29] Hill E.E., Zack E., Battaglini C., Viru M., Viru A., Hackney A.C. (2008). Exercise and circulating cortisol levels: The intensity threshold effect. J. Endocrinol. Investig..

[30] DiNicolantonio J.J., Mehta V., Onkaramurthy N., O’Keefe J.H. (2018). Fructose-induced inflammation and increased cortisol: A new mechanism for how sugar induces visceral adiposity. Prog. Cardiovasc. Dis..

[31] Duong M., Cohen J.I., Convit A. (2012). High cortisol levels are associated with low quality food choice in type 2 diabetes. Endocrine.

[32] Gangwisch J.E., Hale L., Garcia L., Malaspina D., Opler M.G., Payne M.E., Rossom R.C., Lane D. (2015). High glycemic index diet as a risk factor for depression: Analyses from the Women’s Health Initiative. Am. J. Clin. Nutr..

[33] Daniel P.M., Love E.R., Moorhouse S.R., Pratt O.E. (1981). The effect of insulin upon the influx of tryptophan into the brain of the rabbit. J. Physiol..

[34] Inam Q.-A., Ikram H., Shireen E., Haleem D.J. (2016). Effects of sugar rich diet on brain serotonin, hyperphagia and anxiety in animal model of both genders. Pak. J. Pharm. Sci..

[35] Boumosleh J.M., el Hage C., Farhat A. (2021). Sports nutrition knowledge and perceptions among professional basketball athletes and coaches in Lebanon-a cross-sectional study. BMC Sports Sci. Med. Rehabil..

[36] Torres-McGehee T.M., Pritchett K.L., Zippel D., Minton D.M., Cellamare A., Sibilia M. (2012). Sports nutrition knowledge among collegiate athletes, coaches, athletic trainers, and strength and conditioning specialists. J. Athl. Train..

[37] Stone A.A., Schneider S., Harter J.K. (2012). Day-of-week mood patterns in the United States: On the existence of ‘Blue Monday’, ‘Thank God it’s Friday’ and weekend effects. J. Posit. Psychol..

[38] Ryan R.M., Bernstein J.H., Brown K.W. (2010). Weekends, work, and well-being: Psychological need satisfactions and day of the week effects on mood, vitality, and physical symptoms. J. Soc. Clin. Psychol..

[39] Egloff B., Tausch A., Kohlmann C.-W., Krohne H.W. (1995). Relationships between time of day, day of the week, and positive mood: Exploring the role of the mood measure. Motiv. Emot..

[40] Verspeek J., Behringer V., Laméris D.W., Murtagh R., Salas M., Staes N., Deschner T., Stevens J.M.G. (2021). Time-lag of urinary and salivary cortisol response after a psychological stressor in bonobos (Pan paniscus). Sci. Rep..

[41] Odeniyi I.A., Fasanmade O.A., Ogbera A.O., Ohwovoriole A.E. (2015). Body mass index and its effect on serum cortisol level. Niger. J. Clin. Pract..

[42] Schorr M., Lawson E.A., Dichtel L.E., Klibanski A., Miller K.K. (2015). Cortisol Measures Across the Weight Spectrum. J. Clin. Endocrinol. Metab..

[43] Adam T.C., Hasson R.E., Ventura E.E., Toledo-Corral C., Le K.-A., Mahurkar S., Lane C.J., Weigensberg M.J., Goran M.I. (2010). Cortisol is negatively associated with insulin sensitivity in overweight Latino youth. J. Clin. Endocrinol. Metab..

[44] Wurtman R.J., Wurtman J.J., Regan M.M., McDermott J.M., Tsay R.H., Breu J.J. (2003). Effects of normal meals rich in carbohydrates or proteins on plasma tryptophan and tyrosine ratios. Am. J. Clin. Nutr..

[45] Herane-Vives A., de Angel V., Papadopoulos A., Wise T., Chua K.-C., Strawbridge R., Castillo D., Arnone D., Young A.H., Cleare A.J. (2018). Short-term and long-term measures of cortisol in saliva and hair in atypical and non-atypical depression. Acta Psychiatr. Scand..

[46] Bjelland I., Tell G.S., Vollset S.E., Konstantinova S., Ueland P.M. (2009). Choline in anxiety and depression: The Hordaland Health Study. Am. J. Clin. Nutr..

[47] Hibbeln J.R., Northstone K., Evans J., Golding J. (2018). Vegetarian diets and depressive symptoms among men. J. Affect. Disord..

[48] Begdache L., Sadeghzadeh S., Derose G., Abrams C. (2021). Diet, Exercise, Lifestyle, and Mental Distress among Young and Mature Men and Women: A Repeated Cross-Sectional Study. Nutrients.

